# Transdermal Delivery of siRNA through Microneedle Array

**DOI:** 10.1038/srep21422

**Published:** 2016-02-18

**Authors:** Yan Deng, Jiao Chen, Yi Zhao, Xiaohui Yan, Li Zhang, Kwongwai Choy, Jun Hu, Himanshu J. Sant, Bruce K. Gale, Tao Tang

**Affiliations:** 1 Department of Obstetrics & Gynaecology, Faculty of Medicine, The Chinese University of Hong Kong, Shatin, New Territories, Hong Kong, China; 2 Shenzhen Research Institute, The Chinese University of Hong Kong, China; 3 Department of Mechanical and Automation Engineering, The Chinese University of Hong Kong, Shatin, New Territories, Hong Kong, China; 4 Peking University Shenzhen Hospital, Shenzhen, China; 5 State of Utah Centre of Excellence for Biomedical Microfluidics, Departments of Bioengineering and Mechanical Engineering, University of Utah, Salt Lake City, UT 84112, USA; 6 Chow Yuk Ho Technology Centre for Innovative Medicine, The Chinese University of Hong Kong, Hong Kong SAR, China

## Abstract

Successful development of siRNA therapies has significant potential for the treatment of skin conditions (alopecia, allergic skin diseases, hyperpigmentation, psoriasis, skin cancer, pachyonychia congenital) caused by aberrant gene expression. Although hypodermic needles can be used to effectively deliver siRNA through the stratum corneum, the major challenge is that this approach is painful and the effects are restricted to the injection site. Microneedle arrays may represent a better way to deliver siRNAs across the stratum corneum. In this study, we evaluated for the first time the ability of the solid silicon microneedle array for punching holes to deliver cholesterol-modified housekeeping gene (*Gapdh*) siRNA to the mouse ear skin. Treating the ear with microneedles showed permeation of siRNA in the skin and could reduce *Gapdh* gene expression up to 66% in the skin without accumulation in the major organs. The results showed that microneedle arrays could effectively deliver siRNA to relevant regions of the skin noninvasively.

Approximately 20% of known monogenic disorders affect the skin^1^. For the majority of these disorders, there is a lack of effective treatments and there is potential benefit from small interfering RNA (siRNA) therapeutics. The successful development of siRNA therapies has significant potential for the treatment of skin conditions caused by aberrant gene expression, including alopecia^2^, allergic skin diseases^3^,^4^,^5^,^6^, hyperpigmentation, psoriasis, skin cancer^7^,^8^, and pachyonychia congenital^9^. Theoretically, topical siRNA delivery to the skin is relatively easy as it allows direct access to the skin. However, the stratum corneum (the outermost layer of the epidermis) acts as the main barrier to penetration of molecules^10^.

Currently, there are some methods to overcome the barriers in cutaneous siRNA delivery. For example, cell-penetrating peptides have the potential to cross skin barriers, but we should consider the possible toxicity and immunological responses^11^. Still, some physical delivery techniques are also general use, such as cavitational ultrasound, electroporation, iontophoresis or intradermal injection. They are all efficient and effective. However, for the first three methods, complicated equipment or device are required and may be expensive. As for intradermal injection, it is not so “patient-friendly”. Hypodermic needles were used in previous studies for intradermal delivery of therapeutic siRNA, but the injection method caused pain. To provide a less invasive method, the delivery of siRNA into the skin by microneedle devices was investigated^9^.

Microneedle arrays represent a better way to deliver siRNAs across the stratum corneum, and are believed to be less invasive than conventional hypodermic needles^12^,^13^. There are four categories of microneedles in general use; (i) pre-applying solid microneedles to “punch holes”, (ii) incorporating drug into biodegradable microneedles, (iii) coating drugs onto microneedles and (iv) injecting drugs through hollow microneedles^14^. Depending on the available production methods, varied materials have been used for producing microneedles, such as silicon, glass, titanium, ceramic and polymer^15^,^16^,^17^. A line of studies have investigated the delivery of functional siRNA through biodegradable protrusion array device^18^, coated steel microneedles or motorized hollow microneedle array device 19 induced silencing of reporter gene expression in the epidermis of mice. However, there was no report regarding siRNA delivery by solid microneedle.

It is difficult for naked siRNA to enter cytoplasm. Some modifications or carriers could facilitate siRNA taken up by cells, such as cholesterol modification, liposome carrier, nanoparticles, antibodies, aptamers, small molecules, and peptides.

In this work, we evaluated for the first time the capability of a silicon solid microneedle array to punch holes for delivery of the cholesterol modified housekeeping gene (*Gapdh*) siRNA to the skin *in vivo*. This approach utilized an array of fine silicon microneedles delivering siRNA, which should generate little or no pain as the needles do not penetrate sufficiently deep to trigger pain nerve bundles. We hypothesized that this approach could effectively transit the stratum corneum, deposit siRNA into the epidermis and silence the *Gapdh* gene.

## Results

### Microneedles

**Figure 1:**
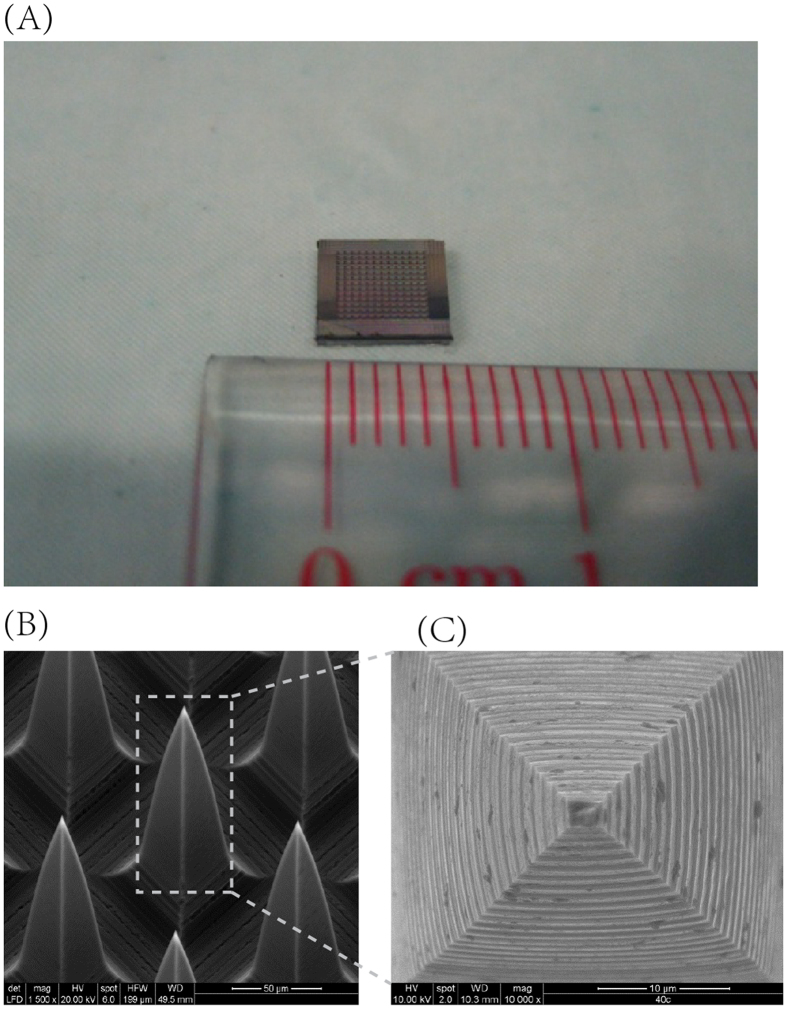
Microneedle array patch. (**A**) The size of the microneedle array patch; (**B**) Configuration of the microneedle array patch and (**C**) Structure of a single microneedle model.

Figure 1 present the SEM image of a typical silicon microneedle array patch, which indicates that the microneedles have uniform morphology and geometry. They exhibit pyramidal shape and the radius of tip is below 1 μm. The length of these fabricated needles is 200 ± 7 μm (n = 900 needles/array and 121 arrays per wafer). The spacing between microneedles is 90 μm with slight variation (i.e. less than 1 μm). For each single microneedle, the surface is rough and layer upon layer.

### Delivery of Cy5-labeled cholesterol siRNA into the epidermis using the microneedles

**Figure 2:**
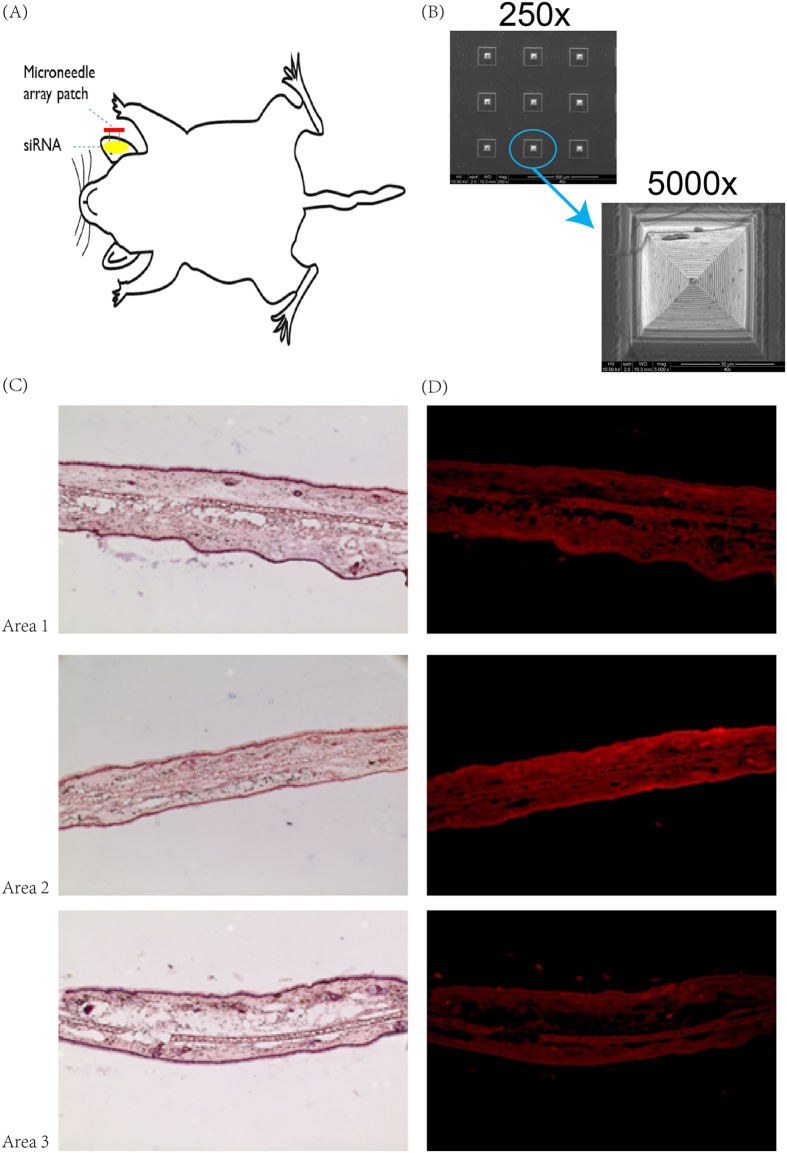
Cy3-labeled cholesterol siRNA distribution in mouse ear skin. Mouse ear skin was treated with Cy3-labeled cholesterol siRNA with microneedle array patch. After six-hour incubation, the ear skin specimen was thoroughly washed, fixed, stained and fluorescently imaged. (**A**) Methods of drug delivery to the skin using microneedle array patch; (**B**) SEM image of microneedle after treatment; (**C**) Sections of mouse ear skin by hematoxylin and eosin staining under optical microscope; (**D**) Sections of mouse ear skin under fluorescent microscope.

We first tested the delivery of Cy5-labelled cholesterol siRNA into the epidermis. The siRNA, to which the skin is impermeable, was placed on the ear skin of mice. And then we pressed the microneedle array patch into the skin for six times (Fig. 2A). The patch then was collected for SEM scanning. Each single microneedle maintained good morphology and geometry (Fig. 2B). Six hours later, the ear skin specimen was thoroughly washed, fixed, stained and fluorescently imaged. As shown in Fig. 2C,D, repeated insertion of microneedles made the skin permeable to siRNA. Skin sections treated with Cy5-labeled cholesterol siRNA exhibit the intense red signal. These images, taken from four separate areas, are representative of all the transverse sections of the analyses samples. In addition, the fluorescent area shows uniform red fluorescence intensity, which may indicate that the microneedles adapted to the tissue profile.

### Distribution of fluorescently Cy5-labeled cholesterol siRNA in mouse by IVIS imaging system

**Figure 3:**
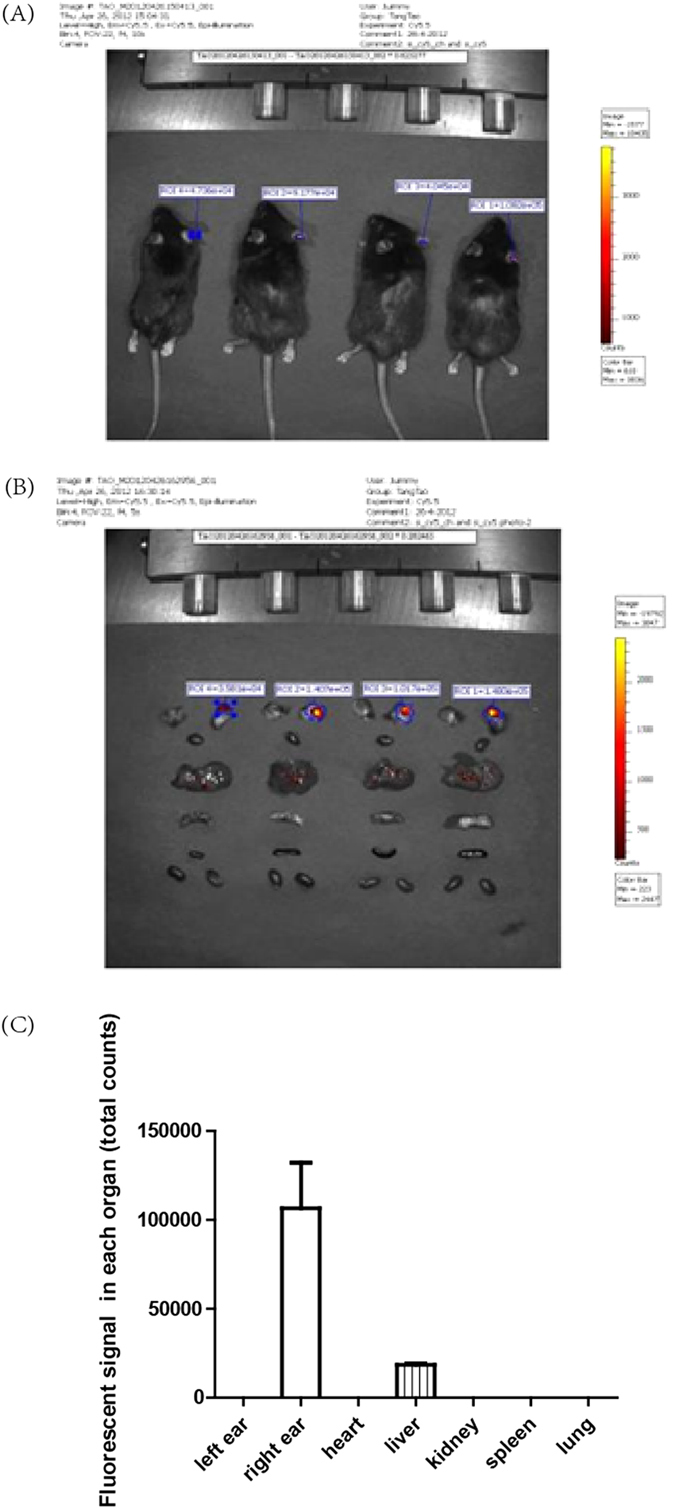
*In vivo* biodistribution of Cy5-labeled cholesterol siRNA in mouse. (**A**) Whole-body fluorescent images; (**B**) Dissected ear, heart, lung, liver, spleen and kidney fluorescent images; (**C**) The quantitative analyses for fluorescent intensity of ear, heart, lung, liver, spleen and kidney.

The *in vivo* biodistribution of Cy5-labeled cholesterol siRNA in mouse was investigated using a non-invasive optical IVIS imaging technique. Whole-body fluorescent images were taken at 6 h after treatment. As shown in Fig. 3, the fluorescence intensity was strong in the treated ear, present in the liver, and absent in the heart, lung, spleen and kidney. In the quantitative analyses, the average fluorescent intensity from the treated ear and liver were 106553 ± 25677 and 18383 ± 961 respectively.

### Silencing of *Gapdh* gene expression

**Figure 4:**
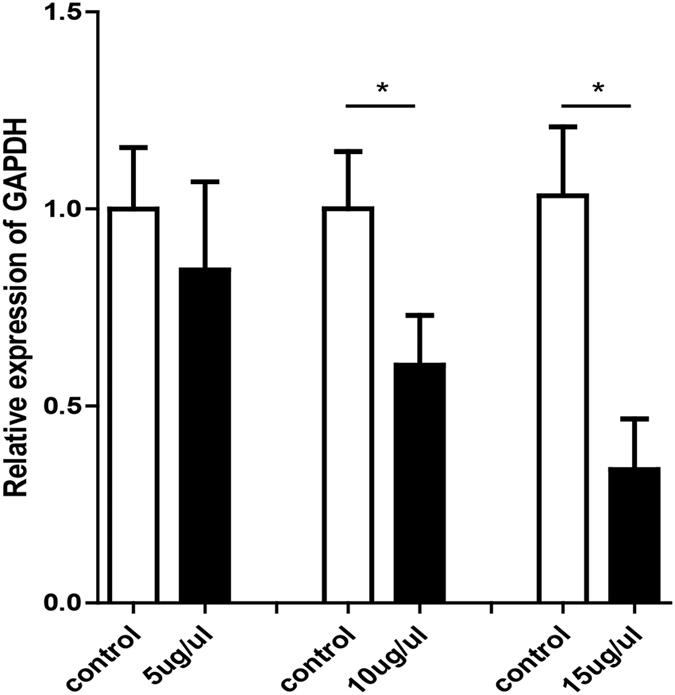
Delivery of siRNA strongly inhibits targeted *Gapdh* gene expression. *P < 0.05, t test.

Mice were terminated at 24 h after the treatment, and the ear skin was excised for RNA extraction. *Gapdh* mRNA levels in the epidermis were measured (Fig. 4). A marked dose-dependent reduction of *Gapdh* gene expression was detected in the skin treated with the siRNA (5, 10 or 15 μg/μl) compared with the contralateral flank skin without treatment. The inhibition percentages are around 15%, 40% and 66%, respectively. The expression of *Gapdh* was normalized by the expression of m-18s.

## Discussion

Gene silencing technology developed rapidly in just a decade. Starting from an academic discovery, it became a potential treatment for human diseases, among which, siRNA is the most promising and has yield the most expectation^20^,^21^,^22^. However, the lack of suitable and effective delivery tools for siRNA is a major barrier to clinical exploitation^23^. The skin has a large surface area and is the most accessible organ of the body. Skin is also an attractive target for siRNA therapies^9^,^18^,^24^,^25^. The siRNA, TD101, targeting the keratin 6a N171K mutant mRNA, has been used for the treatment of pachyonychia congenital. The phase Ib clinical trial has been completed and the safety and efficacy of TD101 was tested. This trial represents the first use of siRNA in human skin^9^,^26^. The observed efficacy appears sufficiently promising to for skin diseases.

At the same time, microneedle arrays have been being developed from the art of fabrication into a science of clinical application over the past 10 years. The initial evolution of microneedles to puncture and create transport channels through the stratum corneum painlessly has been expanded to drug infusion, gene therapy, vaccination and fluid detection systems^27^,^28^. Microneedles have been studied extensively to improve transdermal drug delivery and the possibility of microneedle-mediated agent delivering into the skin has also been confirmed. For example, McAllister *et al.*, treated skin with solid microneedles to permeate latex nanoparticles into human cadaver epidermis. Similarly, Coulman and co-workers showed permeation of polystyrene nanoparticles in human skin by microneedles^29^,^30^. Furthermore, Amit Kumar *et al.* found that pretreatment of mouse skin with microneedles allowed permeation of solid lipid nanoparticles^31^. As expected, in our study, treating the mouse ear with microneedles showed permeation of siRNA in the skin, which is in accordance with the aforementioned results. Since the microneedle surface is rough and layer upon layer, which has a relative large surface area, it can enlarge capacity for siRNA loading. In addition, the SEM image presented here indicated that this type of microneedle array patch can be reused for it can keep the morphology and geometry character.

The delivery efficiency may be a factor for microneedle systems. For the siRNA coated microneedle or biodegradable microneedle, the delivery efficiency can be indirectly measured as follow: the OD reading from coated but unused microneedle arrays provided a total siRNA amount, and was compared to the remaining siRNA from microneedle arrays after application. And the reported delivery efficiency varied from 10% to 85%^18^,^19^,^32^. The delivery efficiency of the microneedles did not translate to gene silencing efficiency. Although our study cannot evaluate the percentage of transdermal siRNA to show the delivery efficiency, a significant silencing efficiency (66%) was observed.

On the other hand, after transiting the stratum corneum barrier, siRNA should also be internalized into cells for functional delivery^33^. However, unmodified siRNAs are not readily taken up by cells in the absence of transfection^34^. Cholesterol and its derivatives have been effectively employed as targeting ligands^35^. It is a useful and common strategy for increasing siRNA’s stability and cellular uptake. Cholesterol-modified siRNA exhibited the prolonged blood circulation time and facilitated siRNA internalization. This enhanced pharmacokinetics resulted in the increase of specific gene silencing efficacy. In a previous study, we have tried to deliver unmodified siRNA by microneedle to the skin and used qRT-PCR to test the knock down efficiency. We have not observed reducing effect of unmodified siRNA. In this study, 3′cholesterol siRNA modifications facilitated cellular uptake *in vivo* without the need for transfection reagent and administration of 3′ cholesterol siRNA by microneedles could reduce *Gapdh* gene expression up to 66% in mouse skin. It was thought that this efficient *Gapdh* gene silencing should be correlated with efficient siRNA internalization.

In order to translate siRNA gene silencing into a therapeutic strategy, it is important to understand the behavior of delivered siRNAs *in vivo*. Usually, it is done by tracing the distribution of siRNA in model animals. For systemic injection, the siRNA molecules are subjected to rapid clearance from the blood through liver accumulation and renal filtration, therefore displaying a patterned distribution in whole-animal imaging assays. In a recent study, naked siRNA was rapidly eliminated from the body through renal filtration and liver excretion, while significant circulatory retention and liver accumulation occurred for the cholesterol-conjugated siRNA, which demonstrated liver targeting of cholesterol-modified siRNA^36^,^37^. Herein, we investigated the fate of locally delivered cholesterol siRNA. Six hours post injection; no siRNA accumulation other than in the treated ear and liver was shown. The fluorescent intensity of ear was much stronger than that of liver. This indicated that siRNAs prefer accumulate in the treated area rather than the major organs comparing with that of systemic administration. It is good for siRNA drug development.

In conclusion, the results serve to demonstrate, for the first time that the solid microneedle arrays could effectively deliver siRNA to relevant regions of the skin *in vivo* noninvasively. It is a proof of concept and this method is simple and cost-effective. Referring to translation to clinical applications, it may offer relief to patients suffering from genetic skin disorders and provide a strong alternative to traditional hypodermic needle injections.

## Materials and Methods

### Fabrication of microneedle array patch

Silicon microneedle arrays were fabricated according to methods described in previous studies^38^,^39^. Briefly, a silicon wafer was diced by a diamond coated dicing blade to create silicon microcolumns or micropillars of required dimensions (height and width) and spacing or pitch. The devices used in this work had a tip-to-tip spacing of 90 μm in both planar dimensions, a designed height of 200 μm, and the blade had a kerf width of 20 μm. A two-step isotropic etching process consisting of dynamic and static etching was employed to achieve sharp tips at the end of the microneedles. The typical etch time was approximate 5 minutes, but specific etch times varied depending on the sharpness of individual tips. If the tips were not completely etched, the etching process was repeated for additional 30 seconds until a sharp tip was obtained for all needles in an array, and 121 arrays of 30 × 30 needles were manufactured simultaneously on each wafer. The etchant was a mixture of hydrofluoric acid (49%) and nitric acid (69%) with a volume ratio of 1:19.

### Animals

Experiments were performed on female C57 mouse (20–22 g) bred and kept by the Laboratory Animal Services Center of the Chinese University of Hong Kong. The methods were carried out in accordance with the approved guidelines.

### Ethics statement

All experiments were performed under license from the Government of the Hong Kong SAR and endorsed by the Animal Experimentation Ethics Committee of the Chinese University of Hong Kong.

### siRNA

The 3′ labeled cy5 and 5′ cholesterol mouse *Gapdh* siRNA was used for imaging and 5′ cholesterol mouse *Gapdh* siRNA was used for gene silencing. All siRNAs was purchased from Tianjin Bestop Biotech Co.,Ltd.

### Delivery of siRNA using microneedle array patch

The mice were anesthetized by Ketamine (75 mg/kg)/Xylazine (10 mg/kg), one ear of each mouse was laid flat on a rigid support. The microneedle array patch was placed in contact with 5 μl of the cholesterol mouse *Gapdh* siRNA solution (5, 10 or 15 μg/μl) on the skin, pressed six times and left unmoved for five minutes. The other ear was used as control.

### Examination of the distribution of Cy5-labeled cholesterol siRNA in ear skin and other organs

The mice were imaged by the IVIS imaging system (Xenogen Imaging Technologies, Alameda, CA) and the siRNA florescence histology analysis (fluorescence microscope) for the distribution of Cy5-labeled siRNA in ear skin was carried out at 6 h after delivery (5 μl of the 10 μg/μl siRNA solution). Subsequently, the mice were euthanized and the major organs (heart, lung, liver, spleen and kidney) were dissected and visualized by IVIS imaging system.

### Hematoxylin and eosin staining

The ear skin was immediately fixed in 10% neutral buffered formalin 6 h after siRNA delivery. Paraffin-embedded tissues were sectioned (20 μm) and stained with hematoxylin and eosin using conventional methods and imaged by standard microscopy.

### Analysis of gene silencing

Mice were euthanized at 24 h after delivery and the ear treated area was isolated for qRT-PCR analysis, the contralateral skin as control. Total RNA was extracted by homogenization in 1 nl Trizol reagent, followed by chloroform extraction and isopropanol precipitation. 100 ng sample of total RNA was reverse-transcribed to cDNA using the commercial protocol. For mRNA amplification, the primers 5′- AGGGCTGCTTTTAACTCTGGT-3′ and 5′-CCCCACTTGATTTTGGAGGGA-3′ were used. qRT-PCR will performed by using Power SYBR^®^ Green PCR Master Mix (Applied Biosystems, Foster City, California). For the analysis of qTR-PCR Data: the relative expression of target mRNA was determined by dividing the target amount by endogenous control amount to obtain a normalized target value. Then the normalized values of the target mRNA were compared among the samples.

### Data analysis

Numeric data in each group will be expressed as mean ± standard deviation and analyzed by t test. Statistical significance is set at *P* < 0.05.

## Additional Information

**How to cite this article**: Deng, Y. *et al.* Transdermal Delivery of siRNA through Microneedle Array. *Sci. Rep.* **6**, 21422; doi: 10.1038/srep21422 (2016).
